# Human leucocyte antigen (HLA-DR) gene expression is reduced in sepsis and correlates with impaired TNFα response: A diagnostic tool for immunosuppression?

**DOI:** 10.1371/journal.pone.0182427

**Published:** 2017-08-03

**Authors:** Martin Sebastian Winkler, Anne Rissiek, Marion Priefler, Edzard Schwedhelm, Linda Robbe, Antonia Bauer, Corinne Zahrte, Christian Zoellner, Stefan Kluge, Axel Nierhaus

**Affiliations:** 1 Department of Anaesthesiology, University Medical Center Hamburg-Eppendorf, Hamburg, Germany; 2 Department of Immunology, University Medical Center Hamburg-Eppendorf, Hamburg, Germany; 3 Department of Intensive Care Medicine, University Medical Center Hamburg-Eppendorf, Hamburg, Germany; 4 Institute of Clinical Pharmacology and Toxicology, University Medical Center Hamburg-Eppendorf, Hamburg, Germany; Charite Universitatsmedizin Berlin, GERMANY

## Abstract

**Background:**

Sepsis is defined as a dysregulated immune response to infection. Impaired immune response in sepsis, often described as endotoxin tolerance, is characterized by unresponsiveness of monocytes on lipopolysaccharide (LPS) stimulation to release tumor necrosis factor α (TNFα). Furthermore, decreased monocyte surface protein expression of human leucocyte antigen DR (HLA-DR) is a marker for changes of the innate immune response during sepsis. Quantitative polymerase chain reaction (qPCR) and flow-cytometry (FACS) have been used to measure protein or gene expression of HLA-DR. We aimed to determine whether changes in mRNA expression of HLA-DR are associated with impaired TNFα response in human sepsis.

**Methods:**

Surface protein together with mRNA expression of HLA-DR were measured by FACS and qPCR in a cohort of 9 sepsis patients and compared to 10 pre-operative control patients in a prospective study. In addition, 20 patients with post-surgical inflammation, 20 patients with sepsis or septic shock were included and TNFα was determined following *ex vivo* stimulation of whole blood with 500 pg/mL LPS. Total RNA was prepared from whole blood and subjected to qPCR analysis for expression analysis of HLA-DR alpha (HLA-DRA) to correlate TNFα response with HLA-DRA expression.

**Results:**

Patients with sepsis presented higher numbers of monocytes in peripheral blood (P<0.001) but decreased surface protein and mRNA HLA-DR levels when compared to controls. In all patients mRNA expression of HLA-DRA was decreased by approximately 70% compared to controls (P<0.01) and was lowest in patients with sepsis or septic shock (P<0.01). TNFα response to LPS was decreased in all patients (median 319 pg/mL versus controls 1256 pg/mL; P<0.01) and lowest in patients with sepsis or septic shock (median 128 pg/mL; P<0.01). HLA-DRA correlated positively with TNFα response in all study participants (r +0.60, P<0.001) and within patients (r +0.67, P<0.001). The TNFα:HLA-DRA ratio correlated negatively with severity and the Sequential Organ Failure Assessment (SOFA) score (Spearman’s rho -0.59, P<0.001)

**Conclusion:**

In this study, HLA-DRA expression was associated with a functional assay of the innate immune response. Future interventional studies aimed at the immune response during sepsis could make use of these methods for optimizing target groups based on biological plausibility and intervention effectiveness.

## Introduction

Sepsis remains the leading cause of death in critically ill patients. It represents a severe and life-threatening response to infection with a loss of immunological homeostasis. In many cases, sepsis leads to a state of hyporesponsiveness, which has been described as “immunoparalysis” or “persistent inflammatory, immunosuppressed, catabolic syndrome (PICS)” with the involvement of both innate and adaptive immune responses [1, 2].

In the past, most therapeutic interventional trials were designed to mitigate different effects of hyper-inflammation. Despite promising phase II studies, hardly any of these interventions could be shown to be beneficial in larger trials [3, 4]. This may be partly due to the fact that (i) circulating soluble biomarkers such as interleukin (IL)-1ß, tumor necrosis factor alpha (TNFα) and IL-6 indicating pro-inflammation have been readily accessible for many years and have been studied extensively [5], and (ii) it has only recently been shown that both pro- and anti-inflammatory activity occur concomitantly during sepsis [6]. However, the tools for the characterization and day-to-day measurement of this dynamic “seesaw” remain largely elusive and existing treatments have limited efficacy.

Among the cellular biomarkers studied in relation to sepsis-induced immunosuppression, monocyte protein surface expression of human leukocyte antigen DR (HLA-DR) has emerged as a reliable parameter representing innate immune function in both experimental and clinical contexts, and has already been used as a promising target in interventional trials [7, 8]. However, flow-cytometric (FACS) determination of HLA-DR is still not widely available and poses technical problems [9]. Cajander et al. found that quantitative real-time polymerase chain reaction (qPCR) analysis of the expression of mRNA encoding a non-polymorphic region of the alpha-chain of the HLA-DR molecule (HLA-DRA) was closely correlated with cell surface expression as measured by flow-cytometry [10].

Moreover, for over a decade, it has consistently been demonstrated in whole blood that decreased TNFα production induced by endotoxin or lipopolysaccharide (LPS) is associated with higher morbidity and mortality in sepsis patients. This observation led to the concept of “endotoxin tolerance” by re-programming of monocytes following the initial exposure to severe infectious stimuli [11, 12]. This hyporesponsiveness correlates with a decreased HLA-DR protein expression, substantiating the hypothesis that whole blood stimulation—if standardized—could serve as a functional assay of the innate immune response [13, 14].

However, the link between a functional assay and the HLA-DRA expression levels in immune cells in whole blood is still missing. In this study, we hypothesize that the mRNA expression level of HLA-DRA, measured by qPCR, is significantly associated with LPS-induced TNFα production in whole blood from patients with sepsis or septic shock.

## Materials and methods

### Study population

From March to December 2014, we enrolled 40 patients (>18 years old) who were admitted to the intensive care unit (ICU) of the University Medical Center Hamburg-Eppendorf (120 beds, Hamburg, Germany). Twenty patients after surgery who were routinely admitted to the ICU for postoperative monitoring were included (group A) and blood was drawn within the first 24h hours after surgery. New admissions to the ICU were screened for the first diagnosis of sepsis or septic shock (group B, n = 20 and validation control group, n = 9). Sepsis was defined as organ dysfunction represented by a SOFA score of 2 points or more. Patients with septic shock were identified by a vasopressor requirement to maintain a mean arterial pressure of 65mm Hg or greater and serum lactate level greater than 2 mmol/L in the absence of hypovolemia [15]. Exclusion criteria were a history of immunological disease, treatment with immunosuppressive medications within the previous 3 months and chronic infection with HI, hepatitis B or C virus. Patients transferred to the ICU from other hospitals were also excluded. After inclusion blood was drawn within 24h and for all patients Sequential Organ Failure Assessment (SOFA) score and Simplified Acute Physiology Score II (SAPS II) score were generated together with routine markers of inflammation: C-reactive protein (CRP), interleukin-6 (IL6), procalcitonin (PCT) and the number of leucocytes. Ten age-matched patients admitted to the hospital for routine surgery with no clinical signs of infection or inflammation were included as controls. Our local ethics committee, the medical chamber of physicians of Hamburg, Germany, approved our study protocol of analyzing the immune response in sepsis patients and controls (protocol PV 4550, including amendment). Informed and written consent was obtained from patients or their legal representatives and all documents and case report files are stored in our ICU study center at the University Medical Centre, Hamburg-Eppendorf. The ethics committee fully agreed to the consent procedure.

### Sampling tubes

Venous blood was drawn within the first 24h after inclusion in all patient groups. Samples were taken at the same time and handled in the same way for FACS, qPCR or stimulation experiments. (i) For FACS analysis in the validation control group EDTA tubes (Sarstedt, Nuembrecht, Germany) were used and samples were processed within 3h. (ii) For mRNA, whole blood was drawn and stored using PAXgene tubes (BD Bioscience, Franklin Lakes, NJ, USA). PAXgene tubes were kept frozen until further analysis. (iii) For the stimulation experiments, whole blood was collected using 4.5 mL lithium heparin tubes (Sarstedt, Nuembrecht, Germany) and stimulated within the next hour.

### Flow-cytometric analysis (FACS)

For phenotypic analysis of monocytes, 100 μl of EDTA blood from controls or sepsis patients was first incubated with IgG (Jackson ImmunoResearch Inc., West Grove, PA, USA) to minimize unspecific antibody binding. For surface staining fluorochrome-conjugated anti-human mAb were added and incubated for 30 min at RT, followed by erythrocytes lysis (Lysing Solution, BD Bioscience, San Jose, CA, USA). The following antibodies were used: anti-CD3 (OKT3) to discriminate T cells, anti-CD19 (HIB19) to exclude B cells, anti-CD56 (HCD56) to separate NK cells, anti-CD14 (M5E2), anti-CD16 (3G8) and anti-HLA-DR (L243) to characterize monocytes. All antibodies were purchased from BioLegend (San Diego, CA, USA). Samples were measured in a FACS LSRFortessa flow cytometer (BD Biosciences, Franklin Lake, NJ, USA) and results were evaluated using FlowJo software (FlowJo, Ashland, OR).

### RNA isolation

mRNA was prepared by using PAXgene Blood miRNA Kit (Qiagen, Hilden, Germany) in accordance with the instructions of the manufacturer. The concentration of mRNA was measured with a NanoDrop ND-1000 Spectrophotometer (Agilent Technologies Germany, Waldbronn, Germany). A ratio of absorbance of approximately 2.0 at 260 and 280 nm was acceptable. The mRNA isolate was stored at −80°C until use.

### cDNA preparation

cDNA was prepared just prior to running the assays. Single-strand complementary DNA (cDNA) was synthesized from 500ng of total mRNA sample with a high capacity with a high capacity cDNA reverse transcriptase kit (Applied Biosystems, FosterCity, CA, USA) in accordance with supplied instructions.

### Gene expression assays

Levels of expression encoding a non-polymorphic region of the alpha-chain of the HLA-DR molecule, HLA-DRA, peptidylpropylisomerase B (PPIB) and cluster of differentiation 14 (CD14) were obtained using quantitative real-time PCR (qPCR). PPIB was used as housekeeping gene together with CD14 in our validation control group. PPIB was used in all patients because of its previously described stability in inflammatory conditions [16]. Briefly, 50ng of cDNA were amplified using an ABI PRISM 7700 System and TaqMan reagents (Applied Biosystems, FosterCity, CA, USA). Validated TaqMan assays were used to assure comparability of serial measurements and a dilution curve was routinely performed. The following primers were used: HLA-DRA (Lot Hs00219575_ml), RefSeq: NM-019111.4, Amplicon 97 base pairs (bp); PPIB (Lot Hs00168719_ml), RefSeq: NM_000942.4, Amplicon 67 (bp) and CD14 (LOT Hs02621496_s1), RefSeq: NM_000591, Amplicon (140 bp). The assays were run in triplicates (20 μL) using water as negative control. Data were generated from 3 separately analyzed plates. For ratio calculation, the average of Ct values for housekeeping genes and HLA-DRA in controls and patients was determined. The delta-delta Ct method was used for normalization. Briefly, we calculated the difference between housekeeping genes and HLA-DRA for each study participant (delta CT). The difference between patient and controls gave the delta-delta Ct. For expression fold change analysis we calculated the 2^-delta-delta Ct as previously described [17].

### LPS-induced TNFα production of whole blood

For stimulation, a 500 pg/mL LPS solution was prepared using LPS from *Salmonella enterica serovar Friedenau* (Glycobiotech, Kuekels, Germany) and stored in pyrogen-free tubes (Sarstedt, Nuembrecht, Germany). A 50 μl blood sample was incubated with 500 pg/mL LPS for 3h at 37°C. Blood was sampled within 1h of withdrawal. After incubation, samples were centrifuged at 1000g for 10 min at 20°C and the supernatant was stored at -80°C for later analysis. TNFα was determined using a solid phase chemoluminescence immunoassay Immulite^®^ on the Immulite^®^ detector system (Siemens Healthcare Diagnostics, Munich, Germany) according to the manufacturer’s instructions.

### Statistical analysis

Differences between groups were tested for significance by using non-parametric Mann-Whitney U test for two groups. Data are presented as medians with interquartile ranges (IQR). Correlations were either calculated using Spearman’s test representing rho and 95% confidence interval or linear regression after transformation of data to reach normal distribution. For all tests, P<0.05 was considered significant. Statistical analyses were performed using Prism 6 (vers. 6 GraphPad Software®, La Jolla, CA, USA).

## Results

Ten preoperative patients with no signs of infection admitted to the hospital for elective surgery served as controls. The patient cohort comprised 20 patients with post-surgical inflammation routinely admitted to the ICU (group A; of these, 14 had undergone major abdominal/ thoracic surgery, 4 orthopedic surgery and 2 neurosurgery), sixteen patients diagnosed with sepsis or septic shock due to underlying medical reasons and 4 for surgical reasons were included in group B (Table 1). The FACS analysis and corresponding qPCR was performed with additional 9 patients with sepsis (validation control group, Table 1). The groups did not differ in terms of sex or age distribution (Table 1). The SOFA and SAPS II scores were used to describe the clinical status. Consistent with this classification, severity scores were higher in group B compared to group A (Table 1).

**Table 1:** Characteristics of study groups.

Categories	Controls	Validation cohort	Group A Post-surgical inflammation	Group B Sepsis and septic shock	P-value Group A vs. B*
Patients, n	10	9	20	20	N/A
Age, years	60 (49–65)	68 (58–74)	62 (47–72)	60 (53–67)	ns
Male/ female, n (%)	8/2 (80/20)	5/4 (55/45)	10/10 (50/50)	9/11 (45/55)	ns
^#^SOFA	N/A	11 (8–13)	4 (3–5)	9 (7–11)	< 0.01
^#^SAPS II	N/A	37 (31–47)	24 (17–28)	32 (27–42)	< 0.01
Death 28d, n (%)	N/A	2 (22)	0 (0)	2 (10)	ns
^#^CRP [mg/L]	5 (5–5)	129 (102–250)	76 (17–109)	183 (143–270)	< 0.01
^#^PCT [μg/L]	N/A	1.17 (0.64–8.80)	0.28 (0.11–1.33)	3.22 (1.26–7.89)	< 0.01
^#^IL-6 [ng/L]	N/A	189 (96–336)	84 (23–195)	308 (85–1080)	< 0.01
^#^Leucocytes x10^9^/L	6.6 (6.1–8.5)	12.0 (8.5–13.5)	13.4 (10.8–15.9)	14.1 (8.0–18.3)	ns

SOFA: Sequential organ failure assessment score; SAPS: Simplified Acute Physiology Score II; CRP: C-reactive protein, PCT: procalcitonin, IL-6: interleukin-6, N/A: not applicable, ns: not significant.

^#^ median (interquartile range, IQR)

* non-parametric Mann-Whitney U test

### Monocytic surface HLA-DR is significantly lower in patients with sepsis

**Fig 1:**
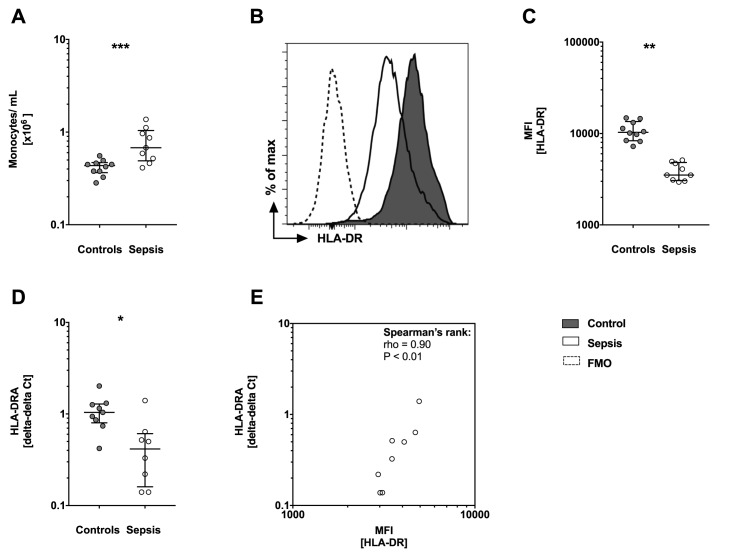
Monocytosis but loss of protein surface and gene expression of human leucocyte antigen DR (HLA-DR) is observed in sepsis patients. (A) Number of monocytes in cells/mL shows significant higher numbers of monocytes/mL in sepsis patients (n = 9) compared to controls (n = 10). (B) Protein surface expression levels of HLA-DR by monocytes are shown as a representative histogram and (C) with the corresponding analysis of median fluorescence intensity (MFI). Sepsis patients show reduced HLA-DR surface expression compared to controls. (A-C) Whole blood was stained for cell surface molecules (CD14 and HLA-DR) and analyzed by flow cytometry (FACS). Data are presented as box and whisker plots with median and interquartile range and statistical analysis was performed using non-parametric Mann-Whitney-U test. (D) Gene expression of HLA-DRA is reduced in sepsis patients. mRNA was prepared from whole blood and HLA-DRA expression levels were assessed by quantitative PCR (qPCR). HLA-DRA expression levels are normalized to internal control gene peptidylpropylisomerase B (PPIB). Data are presented using the delta-delta Ct method and as box and whisker plots showing the median with interquartile range. Statistical analysis was performed using non-parametric Mann-Whitney-U test. (E) Spearman’s rank correlation is presented with median fluorescence intensity (MFI) for HLA-DR protein on the x-axis and mRNA expression of HLA-DR presented as delta-delta Ct on the y-axis. Each circle represents a data set from an individual patient. CD: cluster of differentiation, mRNA: messenger RNA, FMO: Fluorescence Minus One controls, PCR: polymerase chain reaction. P<0.05:*; P<0.01:**; P<0.001:***.

To analyze protein expression whole blood was stained for cell surface molecules. First, neutrophils, NK, B and T cells were excluded. Monocytes were analyzed for expression of CD14, CD16 and HLA-DR in 10 controls and in 9 sepsis patients (validation cohort). The number (cells/mL blood) of monocytes was significantly increased (P>0.05) in sepsis patients compared to controls (Fig 1A). Further, the HLA-DR median fluorescence intensity (MFI) was decreased in sepsis patients (Fig 1B and 1C, P<0.001). Next, we analyzed mRNA expression levels of HLA-DRA in controls and sepsis patients. Corresponding to the FACS data, median mRNA levels of HLA-DRA were significantly decreased in sepsis patients compared to controls, which has been referenced to PPIB (P<0.001, Fig 1D). Further, the HLA-DR MFI correlated strongly with HLA-DRA gene expression in sepsis patients (rho = 0.9, P<0.001, Fig 1E).

### HLA-DRA expression is lower in patients with sepsis

**Fig 2:**
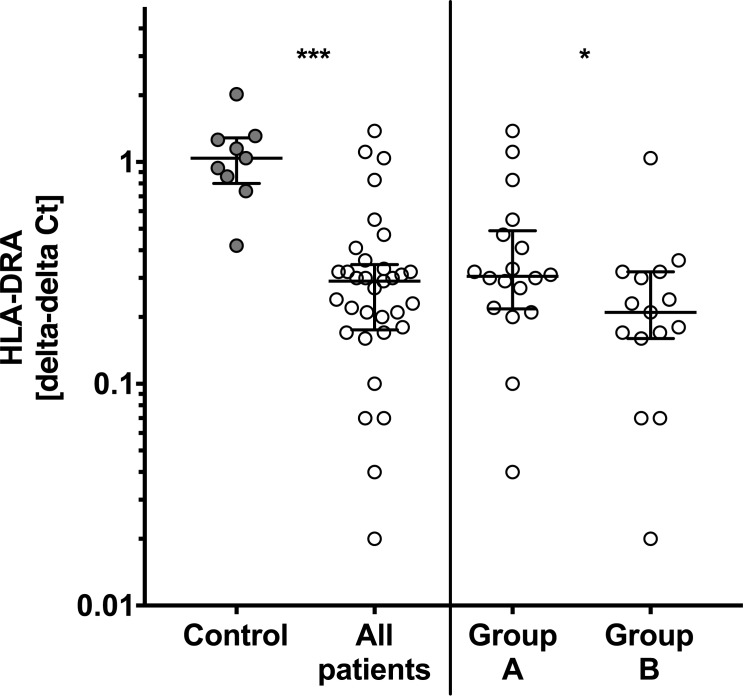
mRNA expression of human leucocyte antigen DR alpha (HLA-DRA) is lowest in sepsis patients. HLA-DRA levels are significant reduced in all patients (n = 40) when compared to controls (n = 10). HLA-DRA levels in patients with sepsis (group B, n = 10) are lowest and significantly reduced when compared to postoperative patients (group A, n = 10). Total mRNA was prepared from whole blood and HLA-DRA expression were assessed by quantitative polymerase chain reaction (qPCR). mRNA levels are normalized to peptidylpropylisomerase B (PPIB) using the delta-delta Ct method. Box and whisker plots with median and interquartile range are presented and statistical analysis was performed using non-parametric Mann-Whitney-U test. P<0.05:*; P<0.001:***.

Whole blood for *ex vivo* stimulation was drawn at the same time as whole blood was collected for mRNA preparation. Mean (SD) ct-value for the housekeeping gene PPIB was 27 (1.5) and no difference was observed between controls and patients. Using the delta-delta Ct method HLA-DRA expression was 70% lower in patients compared to controls (Fig 2, P<0.001). In particular levels were lowest in group B compared to patients in group A: patients with post-surgical inflammation presented a 1.5-fold higher HLA-DRA expression compared to sepsis patients (P<0.05, Fig 2).

### TNFα concentration in stimulated whole blood is associated with patients’ clinical status

**Fig 3:**
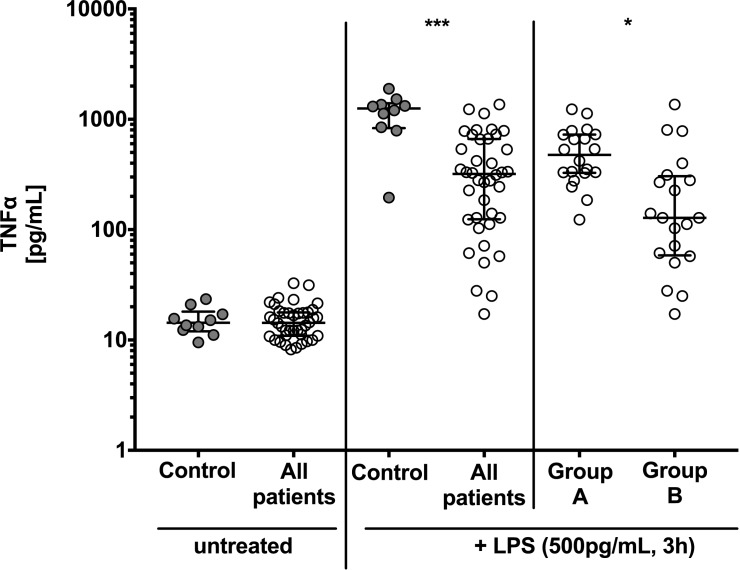
Tumor necrosis factor α (TNFα) response to lipopolysaccharide (LPS) is lowest in sepsis patients. There is no difference in baseline TNFα levels of untreated whole blood in controls and patients. When stimulated with LPS TNFα responsiveness is significant reduced in all patients compared to controls and is lower in sepsis patients (group B) compared to patients after surgery (group A). TNFα levels were determined in the supernatant of untreated whole blood and after stimulation with 500 pg/mL LPS for 3h in controls (n = 10), patients with post-surgical inflammation (group A; n = 20) and patients with sepsis and septic shock (group B, n = 20). Data are presented as box and whisker plots with median and interquartile range. Statistical analysis was performed using non-parametric Mann-Whitney-U test. P<0.05: *, P<0.001: ***.

TNFα was measured in the supernatant of untreated whole blood (baseline) and after 3h *ex vivo* stimulation with LPS. There was no difference in baseline TNFα levels in untreated whole blood between controls and patients (Fig 3). After incubation with 500 pg/mL LPS for 3h, TNFα concentrations were significantly increased in all study participants compared to baseline levels. The median increase was highest in the control group (1256 pg/mL, IQR 830–1400 pg/mL), which was significantly higher compared to all other patients (319 pg/mL, IQR 124–663 pg/mL; P<0.001). In patients with sepsis or septic shock (group B) TNFα concentration was lowest and with 128 pg/mL (IQR 58–304 pg/mL) 3.7-fold lower than in the post-surgical group with 476 pg/mL (IQR: 327–727 pg/mL; P<0.05; group A, Fig 3).

### HLA-DRA expression correlates with TNFα response to LPS

**Fig 4:**
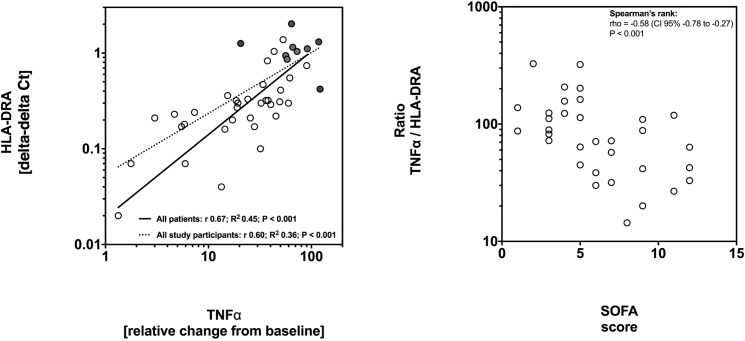
Low expression of human leucocyte antigen receptor alpha (HLA-DRA) is associated with unresponsiveness of whole blood to lipopolysaccharide (LPS) and is associated with disease severity. (A) Whole blood was stimulated with 500 pg/mL LPS for 3h and TNFα levels were determined in the supernatant. The x-axis shows fold changes from baseline TNFα levels after *ex vivo* stimulation. The y-axis shows HLA-DRA expression levels assessed by quantitative polymerase chain reaction (qPCR) using the delta-delta Ct method. Linear regression analysis was performed after transformation to a logarithmic scale to reach normal distribution. Solid line: regression for patients; dotted line: regression for all study participants. Circles represent data from patients and dots represent data from controls. (B) TNFα:HLA-DRA ratio correlates with the SOFA score. Low TNFα response to LPS stimulation is associated with low expression levels of HLA-DRA as shown in (A). The ratio TNFα:HLA-DRA correlates with severity of the disease as measured by the SOFA score. Spearman’s rank test was used to calculate correlation. Circles represent ratios from individual patients.

Next, we performed a regression analysis to determine whether expression levels of HLA-DRA correlate with TNFα response to LPS stimulation. Linear regression analysis revealed a positive correlation between HLA-DRA expression and TNFα levels. The regression coefficient (r) was +0.60 for all study participants (P<0.001) and +0.67 for patients (P<0.001; Fig 4A). To further analyze an association of these findings with clinical severity, the ratio of TNFα to HLA-DRA was calculated. Low ratios were significantly associated with higher SOFA scores, which was significant as determined by Spearman’s test (rho: -0.59 and P<0.001; Fig 4B).

## Discussion

The main findings of our study are that sepsis related monocytosis is associated with loss of surface protein and gene expression of HLA-DR. Moreover, mRNA HLA-DRA expression levels could differentiate between post-surgery and sepsis or septic shock patients. The loss of HLA-DRA was further associated with impaired TNFα responsiveness to LPS.

Our study is in agreement with observed changes during sepsis: Monocytosis with a decreased capacity to produce pro-inflammatory cytokines characterizes immune dysregulation in sepsis [18]. Blood monocytes undergo a functional reprogramming from a pro-inflammatory to an immunosuppressive phenotype contributing to sepsis severity [19]. For clinical use, and to gain insight into the actual state and capacity of the innate immune response, it seems appropriate to move towards a functional test that is meaningful, clinically relevant and readily available.

We were unable to demonstrate significant differences in baseline TNFα levels between controls, patients with post-surgical inflammation or sepsis (Fig 3). During the early phase of sepsis, human monocytes are triggered to produce copious quantities of inflammatory cytokines such as TNFα in response to bacterial pathogens [20]. The median low baseline TNFα levels of only 15 pg/mL (Fig 3) in all study participants can be explained by the well-studied TNFα kinetics: TNFα levels may have already dropped to low levels, which is in agreement with kinetic studies in humans [21]. Considering the rapid changes of TNFα in blood it seems unlikely that measuring absolute concentration of TNFα will be of diagnostic use and a more specific diagnostic tool to evaluate a dysregulated immune system would be of great clinical relevance.

We measured TNFα after incubation of whole blood with *Salmonella enterica*-derived LPS and observed reduced TNFα response in sepsis patients and after surgical trauma compared to controls. Our *ex vivo* results of reduced TNFα levels in sepsis are in accordance with previous clinical studies. For instance, in a cohort of 29 patients, whole blood was incubated with *N*. *meningitides-*derived LPS and decreased production of TNFα was measured: Patients with septic shock could be distinguished from patients with shock due to other causes [22]. Another observation comparing 20 patients after hernia repair or cholecystectomy with 15 sepsis patients showed increased TNFα in the post-surgical group compared to sepsis patients [23]. However, the TNFα response varies between studies and establishing a threshold is challenging. This has been recently addressed by other researchers having shown high variability of TNFα production following stimulation with different LPS sources [24]. In the cited studies LPS from different bacterial sources such as *E*. *coli*, *N*. *meningitides*, *P*. *aeruginosa* and *Salmonella spp*. have been used [22, 23, 25]. Therefore, routinely performed *ex vivo* stimulation of whole blood to identify patients with immunosuppression in sepsis poses practical challenges. Even though most studies have shown that LPS stimulation is associated with decreased TNFα production, we cannot rule out the possibility that different pathogenic factors and different protocols may have affected the results. A reliable, valid and sensitive procedure to diagnose immune suppression would be of great advantage.

Another approach of monitoring the immune response is measuring changes of gene expression. Microarray analysis with over 54000 transcripts in in peripheral blood mononuclear cells from 70 critically ill patients showed that many pro-inflammatory genes are down-regulated [26]. The identification of 138 sepsis “signature” genes could differentiate between sepsis and SIRS patients [26]. The reported alterations in the transcriptome have been associated with alterations in the expression of distinct cell surface markers in immune cells of sepsis patients such as expression of major histocompatibility complex II molecules (HLA-DR). HLA-DR expression changes have been related to an impaired immune response. Antigen presentation by cells of the innate immune system via HLA-DR is a crucial step to develop sustained immune responses to clear the pathogen and reduced presentation of the HLA-DR is a characteristic finding on septic monocytes [18, 27, 28].

HLA-DR is a heterodimer and contains two subunits with HLA-DRA encoding for the α-subunit. We could demonstrate that reduced gene expression of HLA-DR reflects reduced protein surface levels of HLA-DR in patients with sepsis in our validation cohort (Fig 1). In addition, we found decreased mRNA expression levels of HLA-DRA also in a larger cohort of patients (Fig 2).

Numerous studies using FACS analysis to determine HLA-DR levels on monocyte cell surfaces have shown that levels decrease in sepsis. One of these studies showed decreased HLA-DR surface expression in 30 patients with septic shock but not in shock states due to other reasons. Moreover, low HLA-DR levels were an accurate predictor of death in patients with septic shock [29]. These findings are supported by a longitudinal study in 283 ICU patients showing reduced HLA-DR expression levels associated with increased mortality in ICU patients [30].

Without doubt FACS is the current gold standard of measuring monocytic HLA-DR and most research groups have used FACS to analyze patients’ immune status. For validation we prospectively added a small cohort of sepsis patients and compared qPCR results with FACS data (Fig 1). Interestingly in this small cohort of only nine sepsis patients FACS and qPCR reveals the same results: Sepsis patients show lower levels of HLA-DR compared to healthy controls. This observation prompted us to investigate this in a larger cohort of patients and to compare HLA-DRA expression with a functional assay of immune response.

FACS analysis requires an extensive infrastructure and 24h FACS facilities are rarely available in hospitals. Therefore, researchers have hypothesized that changes on the surface levels measured with FACS should be associated with transcriptional changes in mRNA levels. In a recently published prospective trial in 60 sepsis patients the group compared FACS data with mRNA expression data for HLA-DRA [10]. They found that qPCR analysis was robust and reproducible and suggested that this method might be preferable to FACS analysis [10], even though at present PCR would pose a similar problem with respect to laboratory availability. While our findings support previous study results we could further demonstrate that transcriptional changes of HLA-DRA were closely associated also with a stimulated TNFα production.

Last year Drewry *et al*. described a similar approach to identify immunoparalysis in sepsis compared to ours. Whole blood was stimulated with LPS and both TNFα and HLA-DR using FACS were measured in sepsis patients [31]. In accordance to our results TNFα responsiveness and HLA-DR protein expression correlated positively and were lowest in non-survivors of sepsis. Interestingly, the colleagues could not observe differences in TNFα response to LPS between survivors and non-survivors [31]. As mentioned above procedural differences such as the use of different LPS solutions or different time points of stimulation and analysis may explain these results. Importantly HLA-DR is best characterized as a phenotypic marker for severity in sepsis linking the innate with adaptive immunity, whereas TNFα secretion reflects an actual cell function. Besides, reduced TNFα responsiveness as shown may indicates not only monocyte anergy, but also functional impairments of other immune cells such as polymorphonuclear cells and lymphocytes. We show that TNFα:HLA-DRA ratio correlates with severity as measured by the SOFA score (Fig 4B). One may argue that this is an attempt to combine a functional test with a phenotypic immunological marker in sepsis: Measuring both parameters i.e. TNFα release and HLA levels may improve robustness of monitoring immunoparalysis in sepsis.

Nevertheless, our study has several important limitations. (i) It was carried out at a single center and involved only a limited number of patients. The cohort compared elective post-surgery patients with patients admitted to the ICU for sepsis of medical or surgical origin. A larger prospective trial including all patients admitted to the ICU would be of interest. (ii) We did not perform repeated measurements over the whole ICU stay, which might have yielded valuable information about the time-dependent dynamics of HLA-DR expression. (iii) Due to the small sample size, we were unable to show meaningful correlations with other important outcome parameters. (iv) The control group consisted of in-patients admitted for routine surgery and pre-existing immunological abnormalities cannot be fully excluded. (v) We included a validation control group to show that monocytic HLA-DR surface and gene expression levels are reduced independently of monocytes numbers. However, we cannot generalize this finding for all our included study participants. (vi) Since we did not perform a TNFα release assay with our validation cohort, we cannot show that impaired TNFαhat impaired TNFth our validation cohort, we cannot show numbers. patients admitted for routine only a But, since this validation cohort and the patient group B were selected by the same inclusion criteria, we speculate that the reduced HLA-DR expression affects the decreased TNFα. Further, we believe that our PCR methods are robust and have shown a close association with FACS data.

We hope that our observations warrant follow-up studies of larger patient cohorts over a longer period to confirm the power of qPCR measurements to identify immunosuppression.

## Conclusions

In this study, we have shown that qPCR of HLA-DR expression correlates with a functional assay of the innate immune response (LPS whole blood stimulation). Future interventional studies aimed to manipulate the immune response during sepsis could make use of these methods for optimizing target groups based on biological plausibility and intervention effectiveness.

## Supporting information

S1 Table Quantitative summary of the research. (PDF)
