# Supplementary material for: Human leucocyte antigen (HLA-DR) gene expression is reduced in sepsis and correlates with impaired TNFα response: A diagnostic tool for immunosuppression?
Source: PLoS One. 2017 Aug 3;12(8):e0182427. doi: 10.1371/journal.pone.0182427 (PMC5542660; doi:10.1371/journal.pone.0182427)
Supplement: S1 Table — (PDF) [file pone.0182427.s001.pdf]

S1 Table: Quantitative summary of the research

ID	group	Baseline TNFα [pg/mL]	TNFα (+LPS 3h) [pg/mL]	TNFα fold change	mRNA HLA-DRA (rel. Exp.)*	Ratio (TNFα: HLA-DRA)	SOFA score	SAPS II score	CRP [mg/L]	PCT [µg/L]	IL-6 [ng/L]	Leucocytes x 10^9/L
1	control	12.3	1203.0	97.8	N/A	N/A	N/A	N/A	6.0	N/D	N/D	15.0
2	control	15.6	1892.0	121.3	0.42	289.8	N/A	N/A	5.0	N/D	N/D	8.0
3	control	15.1	1359.0	90.0	0.74	121.2	N/A	N/A	6.0	N/D	N/D	10.2
4	control	21.0	1524.0	72.6	1.04	70.0	N/A	N/A	5.0	N/D	N/D	6.1
5	control	9.5	195.0	20.5	1.26	16.3	N/A	N/A	5.0	N/D	N/D	3.6
6	control	13.2	847.0	64.2	2.02	31.8	N/A	N/A	5.0	N/D	N/D	7.5
7	control	23.5	1324.0	56.3	0.94	60.1	N/A	N/A	5.0	N/D	N/D	6.9
8	control	17.1	1125.0	65.8	1.15	57.3	N/A	N/A	5.0	N/D	N/D	6.0
9	control	11.1	1308.0	117.8	1.31	89.6	N/A	N/A	5.0	N/D	N/D	7.0
10	control	13.6	790.0	58.1	0.86	67.4	N/A	N/A	5.0	N/D	N/D	7.3
11	Group A	8.5	665.0	78.2	N/A	N/A	2	16	105.0	0.25	258.9	14.7
12	Group A	8.2	330.0	40.2	0.29	138.1	1	37	5.0	0.06	15.4	22.3
13	Group A	10.8	185.0	17.1	0.20	87.3	1	4	8.0	0.02	9.8	15.1
14	Group A	10.0	331.0	33.1	N/A	N/A	7	19	110.0	0.14	21.1	9.9
15	Group A	9.0	326.0	36.2	0.32	113.7	5	29	71.0	0.55	138.7	10.9
16	Group A	9.2	123.0	13.4	0.04	327.0	2	10	27.0	0.12	13.8	22.7
17	Group A	10.9	538.0	49.4	0.31	156.8	4	23	61.0	0.17	199.1	10.6
18	Group A	12.4	658.0	53.1	1.38	38.5	6	24	106.0	0.80	163.2	10.8
19	Group A	9.6	245.0	25.5	0.21	124.3	3	35	136.0	0.31	142.7	13.6
20	Group A	14.2	532.0	37.5	0.83	44.9	5	25	15.0	0.06	75.2	13.1
21	Group A	13.5	1232.0	91.3	1.11	82.1	3	9	7.0	0.11	12.9	12.9
22	Group A	11.0	352.0	32.0	0.10	321.9	5	24	152.0	2.50	776.0	16.9
23	Group A	16.1	729.0	45.3	0.22	207.3	4	26	21.0	2.98	35.8	23.7
24	Group A	12.1	724.0	59.8	0.30	202.3	5	32	125.0	0.17	92.9	11.9
25	Group A	18.3	1129.0	61.7	0.55	111.4	3	6	86.0	0.09	27.5	13.8
26	Group A	17.6	335.0	19.0	0.27	71.1	6	24	134.0	1.57	182.7	10.9
27	Group A	23.2	786.0	33.9	0.47	72.0	7	31	61.0	1.50	225.2	10.2
28	Group A	16.1	809.0	50.2	0.41	123.3	4	26	80.0	0.05	55.2	10.0
29	Group A	17.4	420.0	24.1	0.33	72.4	3	20	5.0	0.52	64.4	16.1
30	Group A	14.4	278.0	19.3	0.30	63.8	5	27	94.0	0.42	242.2	14.2
31	Group B	13.2	128.0	9.7	N/A	N/A	9	36	383.0	2.82	1582.0	18.5
32	Group B	9.7	140.0	14.4	0.16	89.2	3	23	233.0	21.64	295.2	16.7
33	Group B	10.0	280.0	28.0	0.17	162.1	5	35	78.0	1.23	572.8	12.7
34	Group B	21.1	800.0	37.9	0.32	118.9	11	44	166.0	7.06	321.3	12.2
35	Group B	16.7	50.2	3.0	0.21	14.4	8	35	144.0	0.09	3.6	15.4
36	Group B	18.8	25.0	1.3	0.02	63.5	12	41	273.0	27.40	1182.0	11.2
37	Group B	17.6	313.0	17.8	N/A	N/A	3	27	5.0	1.33	242.8	17.8

**38**	Group B	11.2	61.3	5.5	0.17	31.8	7	27	225.0	1.33	1135.0	5.3
**39**	Group B	12.2	71.4	5.9	0.18	33.0	12	27	297.0	5.70	1298.0	8.7
**40**	Group B	15.8	27.9	1.8	0.07	26.8	11	50	193.0	3.39	458.6	1.0
**41**	Group B	17.8	400.0	22.5	N/A	N/A	7	29	105.0	0.16	31.4	15.9
**42**	Group B	21.5	128.0	6.0	0.07	88.2	9	21	301.0	3.05	69.2	20.4
**43**	Group B	22.0	103.0	4.7	0.23	20.1	9	28	173.0	5.17	70.3	8.0
**44**	Group B	24.1	783.0	32.5	0.30	109.5	9	23	214.0	2.15	914.9	16.6
**45**	Group B	12.2	227.0	18.6	0.32	57.4	7	43	96.0	1.01	643.1	31.8
**46**	Group B	12.2	17.2	1.4	N/A	N/A	11	45	142.0	0.68	254.9	0.2
**47**	Group B	31.4	1362.0	43.4	1.04	41.6	9	28	168.0	25.47	16.4	37.8
**48**	Group B	15.3	112.0	7.3	0.24	30.0	6	24	287.0	8.16	214.9	27.5
**49**	Group B	17.5	269.0	15.4	0.36	42.5	12	42	260.0	5.76	128.3	8.0
**50**	Group B	32.7	57.5	1.8	N/A	N/A	12	42	168.0	75.23	5000.0	3.3
**51**	Validation control	N/A	N/A	N/A	1.40	N/A	9	9	105.0	0.38	40.5	8.3
**52**	Validation control	N/A	N/A	N/A	0.33	N/A	8	43	129.0	1.17	94.0	6.3
**53**	Validation control	N/A	N/A	N/A	0.52	N/A	14	57	35.0	2.02	328.0	12.0
**54**	Validation control	N/A	N/A	N/A	0.14	N/A	11	31	292.0	18.49	392.0	15.4
**55**	Validation control	N/A	N/A	N/A	0.64	N/A	13	47	321.0	0.65	129.6	20.1
**56**	Validation control	N/A	N/A	N/A	N/A	N/A	17	48	100.0	0.73	227.2	13.3
**57**	Validation control	N/A	N/A	N/A	0.50	N/A	3	32	128.0	0.64	97.7	11.3
**58**	Validation control	N/A	N/A	N/A	0.14	N/A	11	35	180.0	15.21	189.5	11.4
**59**	Validation control	N/A	N/A	N/A	0.22	N/A	11	37	215.0	2.40	344.4	14.5

* Total mRNA was prepared from whole blood and HLA-DRA expression levels were assessed by quantitative polymerase chain reaction (qPCR). mRNA levels are normalized to peptidylpropylisomerase B (PPIB) using the delta-delta Ct method

TNFa: Tumor necrosis factor a; HLA-DRA: human leucocyte antigen DR alpha; SOFA: Sequential Organ Failure Assessment; SAPS II: Simplified Acute Physiology Score II; CRP: C-reactive protein; PCT: procalcitonin; IL6: interleukin-6; N/A: not applicable (data not available)
